# Agreement Between AI and Nephrologists in Addressing Common Patient Questions About Diabetic Nephropathy: Cross-Sectional Study

**DOI:** 10.2196/65846

**Published:** 2025-05-02

**Authors:** Niloufar Ebrahimi, Mehrbod Vakhshoori, Seigmund Teichman, Amir Abdipour

**Affiliations:** 1 Division of Nephrology, Department of Medicine, Loma Linda University Medical Center, 11234 Anderson Street, Loma Linda, CA, 92354, United States, +1 9095582624

**Keywords:** artificial intelligence, diabetic nephropathy, nephrologist, ChatGPT, Google Gemini

## Abstract

This research letter presents a cross-sectional analysis comparing the agreement between artificial intelligence models and nephrologists in responding to common patient questions about diabetic nephropathy.

## Introduction

Diabetic nephropathy (DN) is one of the most frequent and severe complications of diabetes, requiring early detection and management [1]. Patients with diabetes should receive accurate information from health care professionals on preventing kidney disease. However, many turn to artificial intelligence (AI) models, like ChatGPT and Google Gemini, for web-based medical information [2-4]. To evaluate the capabilities of ChatGPT-4 and Google Gemini versus nephrologists in providing accurate DN information, their performance in answering the DN-related questions most commonly raised by patients was assessed.

## Methods

### Collection of Questions

To generate patient-focused questions, the following query was prompted to AI models: “What are the most frequently asked questions by individuals regarding diabetic nephropathy?”

The AI-generated responses were systematically reviewed. The final question set was refined and adjusted based on the principal investigator’s experience in clinical practice, ensuring alignment with common patient concerns encountered in real-world practice.

Ultimately, 10 questions covering various DN aspects were developed. Questions 1, 3, and 7 were used to evaluate DN’s diagnosis, risk factors, and prevention, respectively.

Questions 2, 6, and 9 were used to evaluate DN management. Questions 8 and 10 were included to assess DN complications. To evaluate DN progression and severity, questions 4 and 5 were selected.

### Collecting Chatbot and Nephrologist Responses

To ensure consistency, a single investigator entered all questions into ChatGPT-4 and Google Gemini between May 23 and July 7, 2024. Each question was entered into ChatGPT-4 twice—initially and after 45 days—to assess changes in accuracy over time. Google Gemini was used once—concurrently with the second ChatGPT-4 round—and was limited to short-response tasks. Two experienced faculty nephrologists from Loma Linda University with clinical and academic experience also completed the questionnaire via a Google Forms survey.

### Evaluation of Chatbot and Nephrologist Responses

An independent reviewer—a professor of medicine from the same academic center—evaluated AI and nephrologists’ responses. Each answer was graded as “completely inaccurate,” “relatively inaccurate,” “irrelevant,” “relatively accurate,” or “completely accurate.” To prevent grading bias, the reviewer was not informed about the nephrologists’ identities.

### Statistical Analysis

Analyses were conducted by using RStudio (version 4.3.0; RStudio Inc), with *P* values of <.05 considered significant.

### Ethical Considerations

As no patient data were involved, ethical approval was not required. This study adhered to ethical principles for research integrity and transparency.

## Results

Table 1 presents the accuracy distribution of responses for each question assessed by reviewers. No responses were categorized as irrelevant or inaccurate; all were rated as relatively or completely accurate.

Table 2 summarizes the interrater reliability indices among different respondents. The two nephrologists showed statistically significant agreement (κ=0.61; *P*=.04). ChatGPT-4 and Google Gemini had moderate but nonsignificant agreement (κ=0.52; *P*=.10). No significant agreement was found between either AI and the nephrologists (all *P* values were >.05). ChatGPT-4 responses lacked consistency over time (κ=−0.08; *P*=.78). Further analysis showed negligible, nonsignificant agreement among all respondents (κ=0.083; *P*=.41). Excluding ChatGPT-4’s second-round responses did not alter the results (κ=0.09; *P*=.45), confirming the lack of significant agreement.

**Table 1.** Distribution of answers according to each respondent.

Questions	Accuracy of answers				
	ChatGPT-4, first round	ChatGPT-4, second round	Google Gemini	Nephrologist 1	Nephrologist 2
1. What is the gold standard for diagnosis of diabetic nephropathy?	Completely accurate	Completely accurate	Completely accurate	Completely accurate	Completely accurate
2. What is the current standard medication therapy for diabetic nephropathy?	Completely accurate	Completely accurate	Completely accurate	Completely accurate	Completely accurate
3. Can diabetic nephropathy be prevented?	Completely accurate	Relatively accurate	Completely accurate	Relatively accurate	Relatively accurate
4. Can tobacco use accelerate the progression of diabetic nephropathy?	Completely accurate	Relatively accurate	Completely accurate	Completely accurate	Completely accurate
5. How is the severity of diabetic nephropathy determined?	Completely accurate	Completely accurate	Relatively accurate	Relatively accurate	Completely accurate
6. How frequently should a patient be screened for diabetic nephropathy?	Relatively accurate	Completely accurate	Completely accurate	Relatively accurate	Relatively accurate
7. What are the risk factors for the development of diabetic nephropathy?	Completely accurate	Completely accurate	Completely accurate	Relatively accurate	Relatively accurate
8. What is the incidence of kidney failure in diabetic nephropathy?	Completely accurate	Relatively accurate	Completely accurate	Relatively accurate	Relatively accurate
9. When should dialysis begin in diabetic nephropathy?	Relatively accurate	Relatively accurate	Relatively accurate	Relatively accurate	Completely accurate
10. What is the most common cause of death in diabetic nephropathy?	Relatively accurate	Completely accurate	Relatively accurate	Completely accurate	Completely accurate

**Table 2.** Interrater reliability indices^a^ across different respondents.

Respondents	ChatGPT-4, first round	ChatGPT-4, second round	Google Gemini	Nephrologist 1	Nephrologist 2
ChatGPT-4, first round					
κ	—^b^	−0.08	0.52	0.07	−0.08
*P* value	—	.78	.10	.78	.78
ChatGPT-4, second round					
κ	−0.08	—	−0.08	0.23	0.16
*P* value	.78	—	.78	.43	.60
Google Gemini					
κ	0.52	−0.08	—	0.07	−0.52
*P* value	.10	.78	—	.78	.09
Nephrologist 1					
κ	0.07	0.23	0.07	—	0.61
*P* value	.78	.43	.78	—	.04
Nephrologist 2					
κ	−0.08	0.16	−0.52	0.61	—
*P* value	.78	.60	.09	.04	—

a Interrater reliability was measured by using the Cohen and Fleiss κ, with agreement classified as follows: 0.0‐0.20 (none), 0.21‐0.39 (minimal), 0.40‐0.59 (weak), 0.60‐0.79 (moderate), 0.80‐0.90 (strong), and >0.90 (almost perfect) [5].

b Not applicable.

## Discussion

We found that AI models generally provided accurate responses to DN-related questions, with moderate agreement on their accuracy among nephrologists. However, agreement between AI outputs and nephrologists’ assessments was minimal, indicating a lack of standardized evaluation or clinical alignment. Further, the moderate concordance between ChatGPT-4 and Google Gemini suggests similar underlying approaches, and the improved agreement in ChatGPT-4’s second round indicates potential learning and adaptability; however, their limited alignment with nephrologists raises concerns regarding their clinical applicability. Despite that, interactive AI potentially enhances clinical processes by supporting patient education and facilitating communication between patients and clinicians regarding typical disease prevention–related queries [6]; the more questions lean toward subspecialties, the less accurate AI responses tend to be [7].

Although AI models can offer helpful responses about DN, they are not substitutes for thorough clinical discussions, due to observed inconsistencies. Given this study’s preliminary nature, findings should be interpreted cautiously. Further research with larger datasets is warranted to evaluate AI’s reliability in clinical use.

This study has several limitations. The AI models used were not specifically designed for medical applications, and the free versions, which we intentionally selected to reflect typical patient use, may underperform when compared to premium versions. Moreover, including only 2 nephrologists limits the diversity of clinical perspectives, and evaluations by a single senior nephrologist may introduce bias; future studies should include multiple reviewers to strengthen evaluation reliability and validity. Lastly, we did not assess AI responses’ clarity or helpfulness from the patient perspective, highlighting the need for user-centered evaluations in future research.

## Abbreviations

AI: artificial intelligence
DN: diabetic nephropathy

## References

[1] Samsu N (2021). Diabetic nephropathy: challenges in pathogenesis, diagnosis, and treatment. Biomed Res Int.

[2] Miao J, Thongprayoon C, Cheungpasitporn W (2023). Assessing the accuracy of ChatGPT on core questions in glomerular disease. Kidney Int Rep.

[3] ChatGPT — release notes. OpenAI.

[4] Gemini Apps’ release updates & improvements. Gemini Advanced.

[5] McHugh ML (2012). Interrater reliability: the kappa statistic. Biochem Med (Zagreb).

[6] Sarraju A, Bruemmer D, Van Iterson E, Cho L, Rodriguez F, Laffin L (2023). Appropriateness of cardiovascular disease prevention recommendations obtained from a popular online chat-based artificial intelligence model. JAMA.

[7] Caranfa JT, Bommakanti NK, Young BK, Zhao PY (2023). Accuracy of vitreoretinal disease information from an artificial intelligence chatbot. JAMA Ophthalmol.

[8] Certification - ABAIM. The American Board of Artificial Intelligence in Medicine.

